# The Synthesis of Some Perhydrobenzimidazolinium Salts and Their Application in Pd-Carbene Catalyzed Heck and Suzuki Reactions

**DOI:** 10.3390/molecules14062032

**Published:** 2009-06-05

**Authors:** Murat Yiğit

**Affiliations:** Department of Chemistry, Faculty of Science and Art, Adıyaman University, 02040 Adıyaman, Turkey; E-Mail: myigit@adiyaman.edu.tr; Fax: +90 416 223 1774

**Keywords:** Heck and Suzuki reactions, perhydrobenzimidazolinium salt, palladium, carbene, catalyst

## Abstract

Novel 1,3-dialkylperhydrobenzimidazolinium chloride salts were prepared as precursors of *N*-heterocyclic carbenes **3a-e** by reacting *N,N’*-dialkylcyclohexandiamine, triethyl orthoformate and ammonium chloride. The salts were characterized spectroscopically and the complexes formed *in situ* from Pd(OAc)_2_ and **3** have been tested as catalysts in homogenous Heck and Suzuki reactions.

## 1. Introduction

**Scheme 1:**
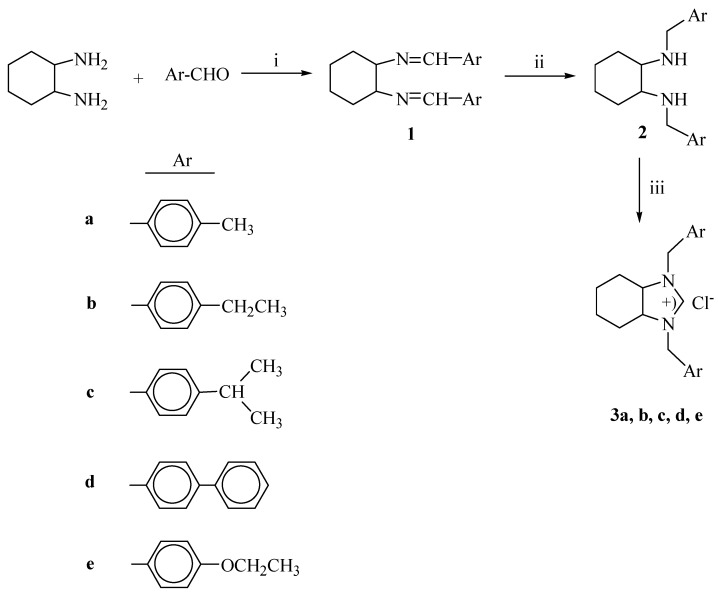
Synthesis of 1,3-dialkylperhydrobenzimidazolinium salts.

Palladium-catalyzed C-C bond formation is one of the most fundemental and important reactions in modern synthetic chemistry [1,2]. It represents the key the step in a wide range of preparative organic processes, from the synthesis of natural products [3] to supramolecular chemistry and material science [4]. Common methodologies used are the palladium mediated coupling of the organic halides or halide equvalents with Grignard reagents, organotin or organoboron reagents where monodentate phosphine are usually employed as ancillary ligands [5,6,7,8]. Recently, *N*-heterocyclic carbenes (NHCs) have been used as alternative or better ancillary ligands than phosphine. The major advantage of NHCs in catalysis is that an increased stability of the active catalyst is often observed, allowing for lower operational catalyst loadings, prolonged reaction times and tolerance of increased reaction temperatures [9,10,11]. The Heck and Suzuki couplings are fascinating reactions from a catalysis science perspective. Virtually all forms of palladium can be used as precatalysts for the simpler reactions, yet specifically designed catalysts are required for activation of bulky or electronically unactivated substrates. Additionally, in many cases, extremely small amounts of palladium (ppm or ppb levels) are sufficient to give very high turnover frequencies, whereas in others, 10% or higher precatalyst loadings are required to obtain adequate product yields [12]. Initially, water-soluble phosphines were used as ligands for the cross-coupling reactions in aqueous media [13], but in recent years, other hydrophilic phosphine-free systems [14] and soluble palladium nanoparticles [15,16,17] have also been found to be higly efficient catalysts for this transformation. The use of water as a solvent for chemical reactions clearly has both economical and environmental advantages because it is inexpensive, abundant, nontoxic, nonflammable and readily separable from organic compounds [18]. There have been a number of reports of the palladium-mediated Heck and Suzuki reaction being performed using water as solvent [19,20,21,22,23,24,25,26,27]. Due to the large number of Suzuki and other coupling reactions that are carried out in aqueous [19] or biphasic systems, there has been an increased interest in the development of water-soluble ligands for these reactions. Shaughnessy and co-workers utilized both sterically demanding water-soluble alkylphosphines [28] and triarylphosphines [29] in Suzuki and Heck couplings of aryl bromides, respectively. We have previously reported the use of the various catalytic systems, such as palladium catalyzed cross-coupling, ruthenium catalyzed hydrogenation and rhodium catalyzed arylation or hydrosilylation [30,31,32,33,34]. Although the nature of the NHC ligand on complexes has a tremendous influence on the rate of catalyzed reactions, the use of perhydrobenzimidazolinium ligands in Heck and Suzuki reactions is a neglected field. In order to find more efficient catalyst, we have prepared a series of new perhydrobenzimidazolinium chlorides, **3a**-**e** (Scheme 1), containing a saturated benzimidazole ring and we report here an *in situ* palladium-carbene based catalytic system for Heck and Suzuki cross-coupling of aryl halides. 

## 2. Results and Discussion

The general route to the *p*-substituent containing ligand precursors is shown in Scheme 1. The condensation of 1,2-diaminocyclohexane with aldehydes in ethanol gave the corresponding Schiff bases in high yields. Reduction of these Schiff bases with NaBH_4_ in methanol leads to *N,N’*-dialkyl-cyclohexan-1,2-diamines. The symmetrical 1,3-dialkylperhydrobenzimidazolinium salts **3** were easly synthesized in high yields from the *N,N’*-dialkylcyclohexan-1,2-diamines, triethyl ortoformate and ammonium chloride. After purification, the 1,3-dialkylperhydrobenzimidazolinium salts **3a-e** were obtained in good yields (85-90%). The salts are soluble in the common polar solvents ethanol and dichloromethane and are stable under air and in the presence of moisture. The structures of **3** were determined by their spectroscopic data and elemental analyses (see Experimental section). The ^13^C- NMR spectra of **3a-e **show only sharp high field signals [159.5 ppm (**3a**), 162.1 ppm (**3b**), 161.9 ppm (**3c**), 162.5 ppm (**3d**), 162.0 ppm (**3e**)] for the imino carbon and for a benzylic carbon [58.7 ppm (**3a**), 66.7 ppm (**3b**), 66.6 ppm (**3c**), 67.0 ppm (**3d**), 66.8 ppm(**3e**)]. The ^1^H-NMR spectra of the perhydro-benzimidazolinium salts further supported the assigned structures. The resonances of the C(2)-H were observed as sharp singlets at δ= 10.87, 10.96, 10.74, 11.10, 10.79 ppm for **3a-e**, respectively. The benzylic protons appeared as a doublet at 4.36- 5.50 ppm. The IR data for perhydrobenzimidazolinium **3a-e** salts clearly indicate the presence of the –C=N- group with a ν_(C=N)_ vibration at 1,644, 1,592, 1,612, 1,587 and 1,600 cm^-1^ for **3a-e**, respectively. These NMR and IR values were similar to those reproted for other 1,3-dialkylperhydrobenzimidazolinium and 1,3-dialkylimidazolinium salts [33,35].

*Reagents and conditions*: (i) EtOH, 3h, reflux; (ii) MeOH, NaBH_4_, 5h, RT; (iii) HC(OEt)_3_, NH_4_CI, 12h, 110 °C.

The Pd-catalyzed Heck reaction is an efficient way to prepare styrene derivatives, which are important chemicals for many applications. The Heck couplings of styrene with aryl bromides to form alkenes were undertaken with Pd(OAc)_2_/**3a-e** as catalysts. The choice of bases and solvents are usually important in achieving an efficient cross coupling reaction. The widely used solvents such as DMF, DMSO, toluene, dioxane, THF and CH_3_CN had remarkable effects on the coupling reaction. For optimal reaction conditions, the Pd(OAc)_2_-catalyzed cross coupling of bromobenzene with styrene was employed as the model reaction using ligand **3a** at 80 ^o^C; Cs_2_CO_3_, K_2_CO_3_, K_3_PO_4_ and *t*-BuOK were tested as the base. The coupling reactions of aryl bromides and styrene were carried out in DMF/H_2_O (3:3 mL) with 1 mol% Pd(OAc)_2_, 2 mol% **3** and 2 equiv. K_2_CO_3_ for 2 h at 80^o^C. The results are summarized in Table 1.

Control experiments indicate that the coupling reactions did not occur in the absence of **3a**. Under these reaction conditions, a wide range of aryl bromides bearing electron-donating or electron-withdrawing groups react with styrene affording the coupled products in excellent yields (Table 1, entries 3, 4, 8, 15, 18 and 22). Enhancements in activity, although less significant, are also observed employing 4-bromobenzaldehyde instead of 4-bromoacetophenone (entries 1–5 and 11-15, respectively).

**Table 1:** The Heck coupling reaction of aryl bromides with styrene. 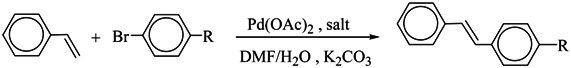

Entry	R	SALT	Yield (%)^b,c^
1	COCH_3_	**3a**	96
2	COCH_3_	**3b**	95
3	COCH_3_	**3c**	98
4	COCH_3_	**3d**	98
5	COCH_3_	**3e**	96
6	CHO	**3a**	95
7	CHO	**3b**	94
8	CHO	**3c**	96
9	CHO	**3d**	91
10	CHO	**3e**	97
11	H	**3a**	94
12	H	**3b**	93
13	H	**3c**	90
14	H	**3d**	92
15	H	**3e**	95
16	OCH_3_	**3a**	92
17	OCH_3_	**3b**	88
18	OCH_3_	**3c**	93
19	OCH_3_	**3d**	91
20	OCH_3_	**3e**	87
21	CH_3_	**3a**	86
22	CH_3_	**3b**	89
23	CH_3_	**3c**	85
24	CH_3_	**3d**	81
25	CH_3_	**3e**	84

*^a^Reaction conditions*: R-C_6_H_4_Br-*p* 1.0 mmol, styrene 1.5 mmol, K_2_CO_3_ 2.0 mmol, Pd(OAc)_2_ 1% (molar ratio), **3a-e** 2% (molar ratio), water (3 mL)/DMF (3 mL), 80°C, 2 h. ^b^Isolated yields are based on aryl bromide. ^c^All reactions were monitored by GC, and the compound purity was checked by NMR.

However, chloroarenes do not react under standard conditions, and yields are typically <5%. A systematic study on the substituent effect in the perhydrobenzimidazolinium salts **3a-e** indicated that the introduction of a *p*-isopropylbenzyl substituent on the *N*-atoms notably increased the yield of product (Table 1, entries 3, 8, 13, 18, 23). Comparatively the perhydrobenzimidazolinium salts displayed better performance than benzimidazolinium salts in the Heck and Suzuki reactions [36,37].

Due to the palladium/imidazolium salt catalyst systems display high reactivity with aryl chlorides in the Suzuki cross-coupling reaction has been applied successfully to the synthesis of fenbuten and to a key intermediate in the synthesis of sartans [38]. The Suzuki coupling of phenylboronic acid with aryl chlorides to form biaryls were undertaken with Pd(OAc)_2 _ /**3a-e** as catalysts.

**Table 2:** The Suzuki coupling reaction of aryl chlorides with phenylboronic acid. 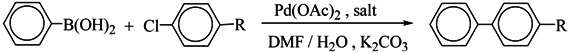

Entry	R	SALT	Yield (%)^b,c^
1	COCH_3_	**3a**	94
2	COCH_3_	**3b**	95
3	COCH_3_	**3c**	97
4	COCH_3_	**3d**	98
5	COCH_3_	**3e**	92
6	CHO	**3a**	93
7	CHO	**3b**	97
8	CHO	**3c**	98
9	CHO	**3d**	94
10	CHO	**3e**	91
11	H	**3a**	87
12	H	**3b**	89
13	H	**3c**	86
14	H	**3d**	94
15	H	**3e**	90
16	OCH_3_	**3a**	84
17	OCH_3_	**3b**	86
18	OCH_3_	**3c**	88
19	OCH_3_	**3d**	89
20	OCH_3_	**3e**	82
21	CH_3_	**3a**	78
22	CH_3_	**3b**	75
23	CH_3_	**3c**	81
24	CH_3_	**3d**	77
25	CH_3_	**3e**	74

*^a^Reaction conditions*: R-C_6_H_4_CI-*p* 1.0 mmol, phenylboronic acid 1.5 mmol, K_2_CO_3_ 2.0 mmol, Pd(OAc)_2_ 1% (molar ratio), **3a-e** 2% (molar ratio), water (3 mL)/DMF (3 mL), 80°C, 1h. ^b^Isolated yields are based on aryl chloride. ^c^All reactions were monitored by GC, and the compound purity was checked by NMR.

Similar reaction conditions were employed for the Suzuki reactions. The coupling reactions of aryl chlorides and phenylboronic acid were carried out in DMF/H_2_O (3:3 mL) with 1 mol% Pd(OAc)_2_, 2 mol% 3 and 2 equiv. K_2_CO_3_ for 1 h at 80^o^C. We started our investigation examining the coupling of 4-chloroacetophenone and phenylboronic in the presence of Pd(OAc)_2 _/**3**. The results are summarized in Table 2. It can be show these salts are an effective ligand precursor for the coupling of unactivated, activated and deactivated chlorides. These results are in agreement with other reports [38,39,40,41,42,43].

## 3. Conclusions

In conclusion, we have synthesized five 1,3-dialkylperhydrobenzimidazolinium chloride salts and have investigated their catalytic activity in the Heck and Suzuki coupling reactions. The procedure is simple and efficient toward various types of aryl halides and does not require an induction period. The advandage of the catalyst is that it has low-loading capabilities, and it is usable in air. Detailed investigations, focusing on imidazolidin-2-ylidene and benzimidazolin-2-ylidene substituent effects, functional group tolerance, and catalytic activity in this and other coupling reactions are ongoing.

## 4. Experimental

### 4.1. General

All reactions for the preparation of 1,3-dialkylperhydrobenzimidazolinium salts **3a-e** were carried out under argon using standard Schlenk-type flasks. Heck and Suzuki coupling reactions were carried out in air. 1,2-Diaminocyclohexane (mixture of *cis* and *trans*), aldehydes and other reagents were purchased from Aldrich Chemical Co. (Turkey). All ^1^H- and ^13^C-NMR were recorded in CDCI_3_ using a Bruker AC300P FT spectrometer operating at 300.13 MHz (^1^H) or 75.47 MHz (^13^C). Chemical shifts (*δ*) are given in ppm relative to TMS, coupling constants (*J*) in hertz. FT-IR spectra were recorded as KBr pellets in the range 400-4000 cm^-1^ on a Mattson 1000 spectrophotometer (wavenumbers, cm^-1^). GC were measured by GC-FID on a Agilent 6890N gas chromatograph equipped with an HP-5 column of 30 m length, 0.32 mm diameter and 0.25 μm film thickness. Melting points were measured in open capillary tubes with an Electrothermal-9200 melting point apparatus and uncorrected. Elemental analyses were performed at TUBITAK (Ankara, Turkey) Microlab.

### 4.2. General procedure for preparation of the 1,3-dialkylperhydrobenzimidazolinium chlorides **3a-e**

A mixture of *N,N’*-dialkylcyclohexan-1,2-diamine (6.80 mmol), NH_4_CI (6.80 mmol) and triethyl orthoformate (5 ml) was heated for 12 h at 110^o^C. Upon cooling to room temperature, colorless crystals of 3a-e were obtained. The crystals were filtered, washed with diethyl ether (3 x 15 mL) and dried under vacuum. The crude product was recrystallized from EtOH/Et_2_O.

*1,3-bis(4-Methylbenzyl)perhydrobenzimidazolinium chloride* (**3a**)*.* Yield: 2.27 g, 90%; mp: 238^o^C; IR (cm^-1^) ν = 1,644 (-CH=N-); ^1^H-NMR (CDCI_3_) δ: 1.32-1.41 (m, 4H, NCHCH_2_C*H*_2_C*H*_2_CH_2_CHN), 1.66-1.69 (m, 4H, NCHC*H*_2_CH_2_CH_2_C*H*_2_CHN), 3.71-3.80 (m, 2H, NC*H*CH_2_CH_2_CH_2_CH_2_C*H*N), 2.29 (s, 6H, CH_2_C_6_H_4_C*H*_3_-*p*), 4.38 and 5.19 (d, 4H, *J*=11.1 Hz, C*H*_2_C_6_H_4_CH_3_-*p*), 7.13 and 7.24 (d, 8H, *J*=6.0 Hz, CH_2_C_6_*H*_4_CH_3_-*p*), 10.87 (s, 1H, 2-C*H*); ^13^C-NMR (CDCI_3_) δ: 21.3 (NCHCH_2_*C*H_2_*C*H_2_CH_2_CHN), 22.8 (NCH*C*H_2_CH_2_CH_2_*C*H_2_CHN), 49.8 (N*C*HCH_2_CH_2_CH_2_CH_2_*C*HN), 18.3 (CH_2_C_6_H_4_*C*H_3_-*p*), 58.7 (*C*H_2_C_6_H_4_CH_3_-*p*), 128.7, 128.8, 130.0, 139.0 (CH_2_*C*_6_H_4_CH_3_-*p*), 159.6 (2-*C*H); Anal. Calcd. for C_23_H_29_N_2_CI: C, 74.89; H, 7.86; N, 7.59. Found C, 74.62; H, 7.71; N, 7.67.

*1,3-bis(4-Ethylbenzyl)perhydrobenzimidazolinium chloride* (**3b**). Yield: 2.41 g; 85%, mp: 146^o^C; IR (cm^-1^) ν = 1,592 (-CH=N-); ^1^H-NMR (CDCI_3_) δ: 1.11-1.48 (m, 4H, NCHCH_2_C*H*_2_C*H*_2_CH_2_CHN), 1.73-2.09 (m, 4H, NCHC*H*_2_CH_2_CH_2_C*H*_2_CHN), 3.10-3.22 (m, 2H, NC*H*CH_2_CH_2_CH_2_CH_2_C*H*N), 1.21 (t, 6H, *J*=7.8 Hz, CH_2_C_6_H_4_CH_2_C*H*_3_-*p*), 2.64 (q, 4H, *J*=7.5 Hz, CH_2_C_6_H_4_C*H*_2_CH_3_-*p*), 4.41, 5.05 and 4.68, 5.26 (d, 4H, *J*=14.7 Hz, and *J*=15.0 Hz, C*H*_2_C_6_H_4_CH_2_CH_3_-*p*), 7.19 and 7.32 (d, 8H, *J*=7.8 Hz, CH_2_C_6_*H*_4_CH_2_CH_3_-*p*), 10.96 (s, 1H, 2-C*H*); ^13^C-NMR (CDCI_3_) δ: 23.5 (NCHCH_2_*C*H_2_*C*H_2_CH_2_CHN), 28.5 (NCH*C*H_2_CH_2_CH_2_*C*H_2_CHN), 50.6 (N*C*HCH_2_CH_2_CH_2_CH_2_*C*HN), 15.4 (CH_2_C_6_H_4_CH_2_*C*H_3_-*p*), 22.6 (CH_2_C_6_H_4_*C*H_2_CH_3_-*p*), 66.7 (**C**H_2_C_6_H_4_CH_2_CH_3_-*p*), 128.6, 130.1, 145.1, 159.4 (CH_2_*C*_6_H_4_CH_2_CH_3_-*p*), 162.1 (2-*C*H); Anal. Calcd. for C_25_H_33_N_2_CI: C, 75.66; H, 8.32; N, 7.06. Found C, 75.24; H, 8.54; N, 7.19.

*1,3-bis(4-Isopropylbenzyl)perhydrobenzimidazolinium chloride* (**3c**). Yield: 2.15 g; 87%, mp: 133-135^o^C; IR (cm^-1^) ν = 1,612 (-CH=N-); ^1^H-NMR (CDCI_3_) δ: 1.09-1.33 (m, 4H, NCHCH_2_C*H*_2_C*H*_2_CH_2_CHN), 1.78-2.04 (m, 4H, NCHC*H*_2_CH_2_CH_2_C*H*_2_CHN), 3.15-3.18 (m, 2H, NC*H*CH_2_CH_2_CH_2_CH_2_C*H*N), 1.20 (d, 12H, *J*=9 Hz, CH_2_C_6_H_4_CH(C*H*_3_)_2_-*p*), 2.85 (hept, 2H, *J*=6.9 Hz, CH_2_C_6_H_4_C*H*(CH_3_)_2_-*p*), 4.66 and 5.50 (d, 4H, *J*=15 Hz, C*H*_2_C_6_H_4_CH(CH_3_)_2_-*p*), 7.18 and 7.29 (d, 8H, *J*=8.1 Hz, CH_2_C_6_*H*_4_CH(CH_3_)_2_-*p*), 10.74 (s, 1H, 2-C*H*); ^13^C-NMR (CDCI_3_) δ: 23.8 (NCHCH_2_*C*H_2_*C*H_2_CH_2_CHN), 27.3 (NCH*C*H_2_CH_2_CH_2_*C*H_2_CHN), 50.5 (N*C*HCH_2_CH_2_CH_2_CH_2_*C*HN), 18.4 (CH_2_C_6_H_4_*C*H(CH_3_)_2_-*p*), 33.7 (CH_2_C_6_H_4_CH(*C*H_3_)_2_-*p*), 66.6 (*C*H_2_C_6_H_4_CH(CH_3_)_2_-*p*), 127.2, 128.5, 130.3, 149.5 (CH_2_*C*_6_H_4_CH(CH_3_)_2_-*p*), 161.9 (2-*C*H); Anal. Calcd. for C_27_H_37_N_2_CI: C, 76.32; H, 8.71; N, 6.59. Found C, 76.81; H, 8.93; N, 6.80.

*1,3-bis(4-Biphenylbenzyl)perhydrobenzimidazolinium chloride* (**3d**). Yield: 2.37 g; 86%, mp: 263-265^o^C; IR (cm^-1^) ν = 1,587 (-CH=N-); ^1^H-NMR (CDCI_3_) δ:1.15-1.28 (m, 4H, NCHCH_2_C*H*_2_C*H*_2_CH_2_CHN), 1.83-2.13 (m, 4H, NCHC*H*_2_CH_2_CH_2_C*H*_2_CHN), 3.26-3.29 (m, 2H, NC*H*CH_2_CH_2_CH_2_CH_2_C*H*N), 4.56, 5.14 and 4.85, 5.37 (d, 4H, *J*=15 Hz and *J*=14.7 Hz C*H*_2_C_6_H_4_C_6_H_5_), 7.33-7.62 (m, 18H, CH_2_C_6_*H*_4_C_6_*H*_5_), 11.10 (s, 1H, 2-C*H*); ^13^C-NMR (CDCI_3_) δ: 23.6 (NCHCH_2_*C*H_2_*C*H_2_CH_2_CHN), 27.5 (NCH*C*H_2_CH_2_CH_2_*C*H_2_CHN), 50.6 (N*C*HCH_2_CH_2_CH_2_CH_2_*C*HN), 67.0 (*C*H_2_C_6_H_4_C_6_H_5_), 127.0, 127.8, 128.8, 129.2, 132.0, 140.1, 141.6, 159.5 (CH_2_*C*_6_H_4_*C*_6_H_5_), 162.5 (2-*C*H); Anal. Calcd. for C_33_H_33_N_2_CI: C, 80.40; H, 6.70; N, 5.68. Found C, 80.21; H, 6.86; N, 5.54.

*1,3-bis(4-Ethoxybenzyl)perhydrobenzimidazolinium chloride* (**3e**). Yield: 1.84 g; 82%, mp: 226^o^C; IR (cm^-1^) ν = 1,600 (-CH=N-); ^1^H-NMR (CDCI_3_) δ: 1.05-1.20 (m, 4H, NCHCH_2_C*H*_2_C*H*_2_CH_2_CHN), 1.70-2.00 (m, 4H, NCHC*H*_2_CH_2_CH_2_C*H*_2_CHN), 3.05-3.07 (m, 2H, NC*H*CH_2_CH_2_CH_2_CH_2_C*H*N), 1.23 (t, 6H, *J*=9 Hz, CH_2_C_6_H_4_OCH_2_C*H*_3_-*p*), 3.91 (q, 4H, *J*=6.4 Hz, CH_2_C_6_H_4_OC*H*_2_CH_3_-*p*), 4.56 and 4.92 (d, 4H, *J*=10.8 Hz, C*H*_2_C_6_H_4_OCH_2_CH_3_-*p*), 6.78 and 7.26 (d, 8H, *J* =8.7 Hz, CH_2_C_6_*H*_4_OCH_2_CH_3_-*p*), 10.79 (s, 1H, 2-C*H*); ^13^C-NMR (CDCI_3_) δ: 23.7 (NCHCH_2_*C*H_2_*C*H_2_CH_2_CHN), 27.8 (NCH*C*H_2_CH_2_CH_2_*C*H_2_CHN), 50.5 (N*C*HCH_2_CH_2_CH_2_CH_2_*C*HN), 14.9 (CH_2_C_6_H_4_OCH_2_*C*H_3_-*p*), 63.6 (CH_2_C_6_H_4_O*C*H_2_CH_3_-*p*), 66.8 (**C**H_2_C_6_H_4_OCH_2_CH_3_-*p*), 115.2, 124.9, 130.1, 159.4 (CH_2_*C*_6_H_4_OCH_2_CH_3_-*p*), 162.0 (2-*C*H); Anal. Calcd. for C_25_H_33_N_2_O_2_CI: C, 70.01; H, 7.70; N, 6.53. Found C, 70.18; H, 7.54; N, 6.71.

### 4.3. General procedure for the Heck coupling reactions

Pd(OAc)_2_ (1.0 mmol%), the appropriate 1,3-dialkylperhydrobenzimidazolinium salt **3a-e** (2 mmol%), aryl bromide (1.0 mmol), styrene (1.5 mmol), K_2_CO_3 _(2 mmol), water (3 mL) and DMF (3 mL) were added to a small Schlenk tube and the mixture was heated at 80 ^◦^C for 2 h. At the conclusion of the reaction, the mixture was cooled, extracted with ethyl acetate–hexane (1 : 5), filtered through a pad of silica gel with copious washing, concentrated and purified by flash chromatography on silica gel. All reactions were monitored by GC. The purity of the compounds was checked by NMR and yields are based on aryl bromide.

### 4.4. General procedure for the Suzuki coupling reactions

Pd(OAc)_2_ (1.0 mmol%), the appropriate 1,3-dialkylperhydrobenzimidazolinium salt **3a-e** (2 mmol%), aryl chloride (1.0 mmol), phenylboronic acid (1.5 mmol), K_2_CO_3 _(2 mmol), water (3 mL) and DMF (3 mL) were added to a small Schlenk tube and the mixture was heated at 80 ^◦^C for 1 h. At the conclusion of the reaction, the mixture was cooled, extracted with ethyl acetate–hexane (1 : 5), filtered through a pad of silica gel with copious washing, concentrated and purified by flash chromatography on silica gel. All reactions were monitored by GC. The purity of the compounds was checked by NMR and yields are based on aryl chloride. 
